# Welcome to American Journal of Medicine Open (AJMOpen)!

**DOI:** 10.1016/j.ajmo.2021.100004

**Published:** 2021-11-15

**Authors:** Stuart R. Chipkin, Joseph S. Alpert

We're proud and excited to announce this innovative forum for communicating new knowledge to practitioners of internal medicine and its related specialties. We are a sibling journal to The American Journal of Medicine. Like any good sibling pair (as adult siblings, not as children), we're going to work to communicate, share, and respect each other while having our own independent existence.

Internal medicine and primary care providers are facing unprecedented challenges that previous generations never had to consider. The direct contact with hospitals or emergency departments is decreasing, but outpatient care manages more severely ill patients than ever before. As specialties increasingly become “sub-specialties”, primary care practitioners are still expected to have a comprehensive knowledge base covering a broad array of fields. As physicians, we remain committed to the ideals of scientific curiosity and a comforting bedside manner, but now we are pushed to adhere to multiple algorithms and flowcharts in order to maximize productivity. While focusing on one patient, we are all being asked to manage populations. It is increasingly challenging to connect the former ideals of care with the new technologies of science and medicine.

## How can a new on-line medical journal help?

First, articles will be totally accessible. AJM Open is an open access journal so no practitioner will be blocked because they do not have a subscription, society membership, or faculty appointment. All of AJM Open's content will be on-line and immediately available so there will be no need to wait for a print version. The editors will seek to use current technology in order to get the word out as quickly and efficiently as possible.

Second, published studies in AJM Open will be relevant to practicing physicians with an emphasis on the realities of outpatient care. As practitioners, we all want to identify health problems before consequences become irreversible. In addition, we want to utilize the best diagnostic tests in the most efficient manner. We need approaches to counsel patients about implementing healthy lifestyle behaviors and making optimal lifestyle choices. When medications are indicated, we need to know outcomes data for benefits in addition to risks and costs. AJM Open articles will be selected for high quality and relevant research to meet these needs.

Third, AJM Open will cross disciplines in the same way that diagnoses do. Our patients usually do not have a single disease in isolation. Instead, they have multiple overlapping conditions presenting as a myriad of clinical symptoms and signs. The impact of our therapies can also cross disciplines–sometimes in positive ways and sometimes with negative outcomes.

AJM Open will seek to publish articles that demonstrate how to extend care beyond the exam room. If we are going to really help patients, we need to increase our awareness of how to assist them after they leave the office. Unfortunately, resources are not always equally available or fairly distributed. Health care professionals should be leaders in their organizations and communities. AJM Open articles will help to identify and advocate for the right services in the right place. Studies demonstrating what works in some neighborhoods should provide support when deciding how to properly allocate limited resources. If some journals aim to reach from “bench to bedside”, AJM Open will seek to reach from “bedside to curbside”.

Advances in technology are giving providers increasingly large amounts of data to evaluate. Data are no longer limited to devices employed in the office. Patients can now keep track of heart rate, blood pressure, EKG, oxygen saturation, blood sugar levels, physical activity, and sleep patterns. More applications will certainly follow. AJM Open will look to publish articles that help separate the most important data signals from background noise. Knowing how best to interpret the potential flood of data from devices can provide opportunities for clinicians to observe how our patients with various medical conditions go about their lives on a daily, and even nightly basis.

AJM Open was founded in order to be a resource for practitioners of internal medicine and its related specialities. Our aim is to have a diverse audience of readers and contributors. We want our readers to be practitioners who are on the front-lines of outpatient medicine and who need new information to provide the best and most cost-effective care to individual patients and to populations of patients in an era that can only be described as “challenging”. We want our contributors to submit high quality research results, reviews, and commentaries that are relevant and generalizable with an emphasis on connecting providers with patients, technology, and community. We look forward to creating a way to assist providers in accessing new knowledge and applying it to their busy professional lives and practices.

Follow us @AjmOpen

About Chronic Diseases | CDC

